# Estimating Neonatal Necrotizing Enterocolitis Based on Feeding Practices

**DOI:** 10.3390/children8040253

**Published:** 2021-03-24

**Authors:** Miguel Baños-Peláez, Valeria Avila-Sosa, Luis Alberto Fernández-Carrocera, Gabriela González-Pérez, Sandra Carrera-Muiños, Maria Antonieta Rivera-Rueda, Guadalupe Cordero-González, Silvia Romero, Alejandra Coronado-Zarco, Estibalitz Laresgoiti-Servitje, Claudine Irles

**Affiliations:** 1 Department of Physiology and Cellular Development, National Institute of Perinatology “Isidro Espinosa de los Reyes”, Mexico City 11000, Mexico; mianbapel@gmail.com (M.B.-P.); valfer2701@gmail.com (V.A.-S.); gonzalezperez.gabriela@gmail.com (G.G.-P.); 2 Neonatal Intensive Care Unit, National Institute of Perinatology “Isidro Espinosa de los Reyes”, Mexico City 11000, Mexico; lfcarrocera@yahoo.com.mx (L.A.F.-C.); sandracarreram@hotmail.com (S.C.-M.); marivera1309@yahoo.com.mx (M.A.R.-R.); guadita69@yahoo.com.mx (G.C.-G.); 3 Neonatal Intermediate Care Unit, National Institute of Perinatology “Isidro Espinosa de los Reyes”, Mexico City 11000, Mexico; silviarmzeta@yahoo.com.mx; 4 Division of Neonatology, National Institute of Perinatology “Isidro Espinosa de los Reyes”, Mexico City 11000, Mexico; irma.coronado@inper.gob.mx; 5 Clinical Sciences, TECSalud, School of Medicine, Tecnológico de Monterrey; Mexico City Campus, Mexico City 14380, Mexico; estibalitz.laresgoiti@tec.mx

**Keywords:** enterocolitis, human milk, neonatology

## Abstract

(1) Background: The relationship between enteral nutrition and neonatal necrotizing enterocolitis (NEC) among premature neonates is still unclear. The present work was designed to assess the relationship between NEC and feeding strategies compared to control infants. (2) Methods: A retrospective case-control study of premature infants (<35 weeks’ gestation) with or without NEC that examined feeding practices and clinical characteristics at birth and 3, 7, and 14-day hospitalization, with a longitudinal and cross-sectional analysis. (3) Results: A total of 100 newborns with NEC diagnosis and 92 neonates without the disease with similar demographic and clinical characteristics were included. The median day of NEC diagnosis was 15 days (Interquartile Range (IQR) 5–25 days). A significantly higher number of neonates that were fasting on days 7 and 14 developed NEC (*p* < 0.05). In the longitudinal analysis, generalized linear and mixed models were fit to evaluate NEC association with feeding strategies and showed that exclusive mother’s own milk (MM) and fortified human milk (FHM) across time were significantly less likely associated with NEC (*p* < 0.001) and that enteral fasting was positively related with NEC. In the cross-sectional analysis, a binary logistic regression model was fit and predicted 80.7% of NEC cases. MM was also found to correlate with a reduced risk for NEC (OR 0.148, 95% CI 0.044–0.05, *p* = 0.02), and in particular, on day 14, several factors were related to a decreased odd for NEC, including birth weight, antenatal steroids, and the use of FHM (*p* < 0.001). (4) Conclusions: MM and FHM were associated with less NEC compared to fasting on days 7 and 14. Feeding practices in Neonatal Intensive Care Units (NICUs) should promote exclusive MM across the two-week critical period as a potential guideline to improve NEC outcome.

## 1. Introduction

Preterm infants are at higher risk of adverse health and developmental outcomes than infants born at term; thus, optimal nutrition and feeding strategies are necessary for this vulnerable population’s adequate growth and development. Necrotizing enterocolitis (NEC) remains the first complication of prematurity, with an annual incidence between 5% and 13% and a mortality up to 30%. Its emergence is inversely related to gestational age and birth weight [1]. Despite multiple studies evaluating its risk factors, the most commonly observed associations continue to be prematurity, enteral feeding, and infection [2]. Several studies have repeatedly shown that maternal milk (MM) offers a protective effect from developing NEC compared to infant formula milk (FM) [3,4]. Experts have recommended MM as the choice of feeding preterm infants, followed by pasteurized donor maternal milk (DM) and FM designed for preterm infants, and they have approved the use of human- or bovine-based milk fortifiers to meet their high nutrient needs [4,5,6,7]. However, there is insufficient evidence regarding the effect of fortification of milk on NEC development, with little or no effect for protection from this disease [3,4,8,9]. 

Concerning feeding strategies, various studies have shown that early trophic feeding, with continuous daily increases compared with prolonging day of starting feeds and slow/intermittent enteral feeding is associated with better weight gain, shorter duration of parenteral nutrition, hospital stay, and reduced incidence of complications such as NEC [5]. It has been reported that delaying and slowing enteral feeding does not reduce NEC’s risk but rather delays weight gain and extends the time to achieve full enteral feeding [10]. Furthermore, the available data from clinical trials do not provide evidence that increasing enteral feeding volumes in daily increments of 15 to 20 mL/Kg (compared to 30 to 40 mL/Kg) reduces NEC or risk of death in very premature infants [11]. An increased number of days fasting was associated with NEC [12] and surgical NEC (SEC) [13].

To date, only few studies have analyzed feeding schemes across time and its association with NEC. In a cohort study by Johnson and cols, the authors analyzed daily volume and percentage of maternal milk, FM, and fortified human milk (FHM) exposure during a period of 14 days, and they found that FM in this period increased the risk of NEC 3.5 times in Very Low Birth weight infants (VLBW) [14]. A retrospective study of 349 VLBW infants evaluated the intake of MM and FM during the first 10 days [15]. However, there is still limited evidence in the incidence of NEC with different feeding strategies analyzed from a longitudinal and cross-sectional point of view in the early period after birth [16].

Our work aimed to elucidate NEC’s association in premature infants <35 weeks gestational age with feeding strategies at birth, three, seven, and fourteen days of life, taking into account clinical and demographic variables.

## 2. Materials and Methods

### 2.1. Ethics

The protocol was approved by the Institutional Research and Ethics Committees of the National Institute of Perinatology “Isidro Espinosa de los Reyes” (INPer) (Project numbers: 2017-2-5 and 2018-149-1) and conducted in accordance with the Declaration of Helsinki. No consent form was required to retrieve information from electronic medical records since this was an analysis of de-identified data.

### 2.2. Patients

Infants were selected based on the following eligibility criteria: gestational age less than 35 weeks, adherence to receive feeding protocol on the basis of the use of maternal milk, and initiation of enteral feeding before 7 days after birth. Infants with congenital anomalies, chromosomopathies, and suspected metabolic disorders were excluded, as well as those with spontaneous intestinal perforation (SIP). SIP was diagnosed and differentiated from surgical NEC (SEC) according to clinical and histopathological criteria [17]. Briefly, pneumatosis, portal venous gas, and necrosis of the mucosa are absent in SIP. Infants were categorized into NEC or non-diseased groups (case/control study). NEC was diagnosed by an expert clinical team according to the presence of clinical and radiographic criteria fulfilling modified Bell’s stage ≥2 and ≥3. Infants with a diagnosis of Bell stage 1 were excluded from the study. Control infants were paired with case infants matched for gestational age and birth weight and selected based on temporal proximity to NEC cases and baseline/early clinical and demographic characteristics. Comparison of baseline variables between Control and NEC infants are listed in Table 1 and Table A1 (statistical tests in Appendix A).

### 2.3. Database

The database with clinical, anthropometric, and feeding information of eligible infants born less than 35 weeks gestational age was obtained from medical records of infants admitted to the NICU of the INPer from 2015 to 2019 and retrospectively analyzed. In this period of time, a total of 649 preterm infants (<35 week’s gestation) were hospitalized in this health center, with an average incidence of NEC of 15.4%. Clinical and feeding information was extracted from medical records at birth and days 3, 7, and 14 by a neonatologist and specialized nurses. The primary study exposures were determined as continuous and multilevel categorical variables.

### 2.4. Feeding Practices

This level 3 health center is a national referral center for Perinatology admitting only high-risk pregnancies and follows Institutional Guidelines for Neonatology based on current literature. Feeding recommendations are the following. The first, second, and third choices of milk are mother’s own milk (MM), pasteurized donor milk (DM), and preterm formula milk (FM), respectively. The criteria for switching MM or DM to FM is that MM or DM are unavailable. Trophic feed is started within 24 h of birth after confirming that abdominal examination is normal and the neonate is vigorous. Feeding is only contraindicated if there is an intestinal obstruction or abdominal distension. Increases in milk volume for neonates under 1500 g are 12.5 mL/kg/day and for >1500 g of 25 mL/kg/day. Fortified human milk (FHM) with a bovine-based milk fortifier was initiated after attaining enteral intake of 100 mL/kg/day at a concentration of 1:50.

Different feeding regimens for birth and days 3, 7, and 14 were identified, and the following categorical variables (6 categories) were created for each day: enteral fasting (Fasting), exclusive MM, exclusive DM, exclusive FM, or the combination of MM with DM or FM (MM + DM or MM + FM, respectively), and FHM. Infants receiving MM could switch to another group, and this was taken into account by the categorical variable which contains all groups of feeding strategies and by the longitudinal analysis of the data across all time points.

From clinical and demographic variables, the following data were extracted at birth: the number of doses of antenatal steroids (none, one or two doses or more), intrauterine growth restriction (IUGR, yes/no), chorioamnionitis (yes/no), infant sex (female/male), maternal age (years), gestational age at birth (weeks), birth weight (g), premature rupture of membranes (PROM, yes/no), surfactant (yes/no), histamine H2 receptor block (yes/no), antenatal antibiotics prior to birth (yes/no), maternal infection prior to birth (yes/no), proton pump inhibitor (yes/no), Continuous Positive Airway Pressure or CPAP (yes/no), endotracheal intubation (yes/no), sepsis (no, early-or late-onset), Patent Ductus Arteriosus (PDA, yes/no), and Infant Respiratory Distress Syndrome (IRDS, yes/no). In addition, day of NEC diagnosis, first day of enteral feeding, volume of first enteral feed, and daily increases were also obtained. All data were imported into Microsoft Excel.

### 2.5. Statistical Analysis

Descriptive statistics were reported as Median (Interquartile Range, IQR) for continuous variables (gestational age, birth weight, maternal age, day of NEC diagnosis, first day of enteral feeding, volume of first enteral feed, and daily increases), or frequencies (%) for categorical variables (IUGR, antenatal steroids dose, feeding regimen, PROM, surfactant, chorioamnionitis, histamine H2 receptor block, antenatal antibiotics, maternal infection, proton pump inhibitor, CPAP, endotracheal intubation, early- and late-onset sepsis, PDA, and IRDS). All numerical variables were assessed for normality using the Shapiro–Wilk Test and the presence of outliers. This case-control study’s main outcome was the estimation of NEC (Cases) or no disease (Controls) based on feeding practices and clinical variables. Controls were selected and paired with cases according to gestational age and birth weight. The Mann–Whitney U test and Student’s *t*-test were used to compare two means of numerical non-normally and normally distributed variables, respectively. The frequencies of categorical variables were analyzed using Pearson’s or Fisher’s exact χ2 tests. For each time point, the *p*-value corresponds to the comparison between Control and NEC infants in the overall χ2 test among the categorical variable feeding regimen. However, each variable had seven feeding groups, and the comparison for these distinct types of feeding regimens between Ctrl and NEC was based on a z-test for independent proportions (adjusting the *p*-values with the Bonferroni method). Within each feeding regimen, the possible pair of percentages between Control and NEC is compared using a z-test. When the result was significantly different, this was denoted as the * symbol (*p* < 0.05).

### 2.6. Regression Models

For the longitudinal analysis across three time points at days, 3, 7, and 14, the data were in the long format. A generalized linear model (GLM) with a linear link function and a mixed model were fit for NEC dichotomous variable with feeding variables (MM, DM, FHM, and fasting) and antenatal steroids doses received and adjusted for gestational age at birth, birth weight, and IUGR. SAS Studio was used to perform the GLM model. This analysis will diminish the potential reverse causality. The cross-sectional analysis with the data in the wide format was performed with binomial logistic regression models fit to evaluate if feeding variables such as fasting, type of milk at days 3, 7, and 14 and antenatal steroids doses received were associated with NEC adjusted for gestational age at birth, birth weight, and IUGR. The data were analyzed using SPSS, version 26.0 (SPSS Inc., Chicago, IL, USA). A value of *p* < 0.05 was considered significant.

## 3. Results

### 3.1. Descriptive Statistics

A total of 192 newborns were included in this control-case study. Of these, 92 (48%) were neonates who attended NICU without a diagnosis of NEC, and 100 infants were diagnosed with enterocolitis (52%), of which 32 were surgical enterocolitis (SEC) (16.4%) (Table 1). The median for NEC diagnosis was 15 days (IQR, 5.2–25 days), with a range between birth and 97 days. NEC onset’s cumulative frequencies were the following: 31 neonates developed NEC in the first seven days, 19 neonates developed NEC between 7–14 days, 19 neonates developed NEC between 14–21 days, 14 neonates developed NEC between 21–28 days, and 17 neonates developed NEC after 28 days. Only three neonates presented NEC at birth.

Control and NEC infants had similar demographic characteristics (maternal age, gestational age, birth weight, infant sex) and baseline clinical variables associated with NEC such as antenatal antibiotics, intrauterine growth restriction, chorioamnionitis, PROM, surfactant, and early mechanical ventilation, such as CPAP or endotracheal intubation (*p* > 0.05; Table 1 and statistical tests in Table A1). Regarding other risk factors for NEC, maternal infection and antenatal steroids were significantly increased in control infants compared to NEC infants (54.3% vs. 35% and 84.8% vs. 63.4%, respectively; *p* < 0.01, Table 1 and Table A1). No significant differences were found for early- and late-onset sepsis, PDA, and proton pump inhibitor use, although sepsis was increased in NEC infants (Table A2). A significantly decreased number of NEC infants presented Infant Respiratory Distress Syndrome (38% vs. 57.6%) and were administered Histamine Receptor H2 block (*p* < 0.01, Table A2).

When comparing birth weight between groups (Table 2), a significantly lower percentage of infants that developed NEC had a birth weight > 1500 g compared to control infants (15% vs. 35%, *p* < 0.05), whereas a higher number of NEC neonates had a birth weight of < 1500 g in comparison with infants without NEC.

#### Inferential Statistics

A crosstab analysis was performed to compare the type of feeding at birth and days 3, 7, or 14 after birth in both groups of infants (Table 3). Significant differences were found between Control and NEC infants in the type of feeding at days 7 and 14 (*p*= 0.013 and *p* < 0.001, respectively), but not at birth (*p* = 0.237) nor day 3 (*p* = 0.806).

A significantly higher number of NEC infants were fasting at days 7 and 14 than controls (32.3% vs. 11.2%, *p* < 0.05, and 31.6% vs. 4.9%, *p* < 0.05, respectively). Sixty one percent of babies in the control group were exclusively fed with exclusive MM at day 7 compared to 36.4% of infants diagnosed with NEC (*p* < 0.05). Similarly, 53.8% of control group newborns received exclusive MM at day 14 compared to 37.8% in the NEC group (*p* < 0.05). Twenty control infants were receiving HMF at day 14 compared to 11 neonates who developed NEC (*p* < 0.05).

In all feeding groups, several neonates received a combination of MM with DM or FM, but no significant between-group differences were found at birth, at day 3, at day 7, nor day 14; the number of neonates under this feeding scheme was minimal (around 4–11%).

Regarding the reasons for fasting, feeding is contraindicated in infants with vomit, Patent Ductus Arteriosus (PDA), shock, and due to the development of NEC. It is important to mention that eight infants were fasting due to NEC at day 7 and 1 infant at day 14; thus, NEC fasting corresponds to the 25% of the fasting group at day 7 and 3% at day 14 (8/32 and 1/31 fasting infants due to NEC at days 7 and 14, respectively).

Regarding feeding practices such as time of starting and volume, there were no significant differences in the first day of enteral feeding and volume increases; however, control infants had a higher volume of first enteral feed than NEC infants (21.2 ± 18.7 mL vs. 15.5 ± 10.3, *p* < 0.05). Newborns that developed NEC were fed earlier than the infants with SEC (1.9 ± 0.4 vs. 2.9 ± 3.2, *p* < 0.05) (Table 4).

### 3.2. Regression Models

For the longitudinal analysis, we fit a generalized linear model (GLM, Table 5) and mixed model (Table A3) considering three time points (days 3, 7, and 14). The GLM model was significant (*p* < 0.001, AIC 664, BIC 716), showing that two doses and one dose of antenatal steroids were negatively related with NEC (estimates -0.188 and -0.414, respectively). In addition, the model showed that newborns with a birth weight >1500 g, and those within the range of 1000 g to 1500 g had a significantly increased association with NEC compared to those with less than 1000 g (estimates -0.478 and -0.143, respectively). Infants of 28–32 and 32–35 week’s gestational were significantly more likely to develop NEC in comparison with those born at 25–28 weeks (0.164 and 0.166, respectively). Newborns receiving MM and FHM across the three time points were significantly less likely associated with NEC (estimates -0.117 and -0.124, respectively). On the other hand, enteral fasting was positively related with NEC (estimates 0.148). We found similar results with the mixed model (Table A3).

Then, a cross-sectional analysis with binary logistic regression was fit to evaluate if feeding regimen (fasting, type of milk received) collected at day 3, day 7, and day 14 were associated with NEC (Table 6 and Table A4). The model was statistically significant (*p* < 0.001) with a medium effect size (Nagelkerke R2 = 0.508) and correctly predicted 80.9% of cases. The regression showed that the administration of two doses of prenatal glucocorticoids significantly reduced enterocolitis development (AdjOR = 0.157, 95% CI 0.052–0.474, *p* = 0.001). No significant differences were found on day 3 (Table A4) and on day 7, only MM was associated with a decreased number of NEC cases (AdjOR = 0.148, 95% CI 0.044–0.501, *p* = 0.02). On day 14, infants who received exclusive MM, a combination of MM and DM, or FM showed a lower risk of NEC compared to control newborns (AdjOR = 0.048, 0.060, and 0.033, respectively). As well, a significantly reduced odd for NEC was observed in newborns with HFM on day 14 (AdjOR of 0.027, 95% CI 0.004–0.179, *p* < 0.001). No associations were observed for those fed with DM (*p* = 0.438) or FM combined with MM (*p* = 0.82) (Table A4).

Logistic regression analyses were also fit to evaluate Ctrl and NEC’s association at day 3, day 7, and day 14, independently (Table 7 and Table A5). No significant associations for feeding schemes were found on day 3 (*p* = 0.899, Table A5). However, the regression model performed solely with day seven or day 14 variables revealed similar results as those seen previously. Exclusive MM at day seven and day 14 individually was significantly related to decreased NEC development (AdjOR = 0.180, 95% CI 0.070–0.462, *p* < 0.001, AdjOR = 0.068 95%CI 0.017–0.269, *p* < 0.001). These models were statistically significant (*p* < 0.001, Nagelkerke R2 = 0.359 and *p* < 0.001, Nagelkerke R2 = 0.424), and correctly classified 73.8% and 72.5% of cases, respectively. On day 14, only DM was not associated with a decreased NEC risk compared with other feeding schemes (*p* = 0.589, Table A5).

Finally, we compared the feeding regimen and NEC onset before or after 7 days (Table 8). In the group that presented NEC before day 7, a statistically higher number of newborns were fasting (*p* < 0.001). However, on day 14, more neonates received human milk (42%) who developed NEC after day 7, although the sample was small. Concerning the other types of feeding, there were no significant differences.

## 4. Discussion

The importance of enteral feeding in preterm infants has been extensively studied, but there are still conflicting data on the impact of feeding on NEC development. We aimed to analyze enteral feeding schemes and its association with NEC across time (birth and days 3, 7 and 14). We found that exclusive mother’s own milk (MM) and fortified human milk (HFM) across days 3, 7, and 14 were negatively associated with NEC in contrast to enteral fasting that was positively related with the disease. In a cross-sectional analysis at day 3, 7, or 14, MM was found to correlate with a reduced risk for NEC on day 7 and 14 compared to fasting. On day 14, several factors were related to a decreased odd for NEC, including birth weight, antenatal steroids, and the use of FHM.

Feeding preterm neonates is still controversial; in some cases, enteral feeding appears to protect against the onset of NEC, and in others, it promotes changes that may contribute to the disease [3], although several guidelines have been published for VLBW infants [3,4].

Although we found no statistically significant differences between neonates that developed NEC vs. control infants in gestational age or IUGR in our study, it has been reported that more than 85% of NEC cases are less than 32 weeks’ gestation [18,19]. We also found that NEC cases in this study have a mean of 30 weeks and this is also in accordance with a study of Sharma and cols. [20]. Birth weight is another well-known factor negatively associated with NEC. In this study, weight at birth for the NEC group was lower than for controls <1200 g, which is in accordance with the literature showing that NEC occurs in 11–15% of those neonates who weigh less than 1000 g and in 4–5% of those between 1001 and 1500 g [20].

Most cases of NEC (>95%) develop after enteral feeding begins, usually in the second week of life (8–10 days), when they receive an enteral supply of 100–120 mL/Kg/day; although in preterm infants <29 weeks, the initial clinical picture occurs later, with a mean age of 14–27 days [21]. In this study, the median number of days of NEC diagnosis was 15 days, which is one week more than that reported by Gasque-Góngora and cols. [11]. Moreover, NEC has also been categorized according to its onset as early-onset (<7 days) and late-onset (>8 days) by some authors [22]. Some other factors may intervene in our study [23].

One of the few well-established obstetric interventions to treat pregnant women at risk for preterm delivery is antenatal steroid administration to improve perinatal outcomes [24]. A review of the literature report on the benefit of prenatal steroids to reduce infant respiratory distress syndrome and a trend for a diminished association with NEC [25] and advanced NEC was associated with reduced antenatal steroids compared to controls in a study in a multivariate regression model [26]. These results are in accordance with our results showing a statistically significant benefit of steroids in decreasing NEC risk, further extending its implication with administering two doses.

Regarding the first day of enteral feeding in this study, there was a tendency to delay feeding in patients who developed NEC, without reaching significance. Although the increases in milk volume were on average higher for neonates that presented NEC, there was no statistical difference. In contrast, our results demonstrate that prolonged fasting across time was associated with NEC in generalized linear and mixed models, which has been mentioned as a risk factor in the literature. In our NEC cases, a significantly higher number of infants who were fasting on days 7 and 14 developed NEC compared to controls. A multicentric study of the Canadian Neonatal Network showed that neonates who developed NEC were fasting on average 5.6 days in contrast with 3.7 days in controls [12]. Likewise, in this study, the GLM, mixed, and logistic regression models showed a significant decrease in the risk of NEC with exclusive feeding of MM across time, and in particular at 7 and 14 days. It was also shown that all diet types and their combinations compared to fasting had a protective factor. However, this is in contrast with results from a meta-analysis showing no statistically significant differences in the incidence of NEC with prolonged enteral fasting compared with early trophic feeding (meta-analysis, risk ratio 1.07 (95% confidence interval 0.67 to 1.70) [27]. In a retrospective study of VLBW infants, the authors showed in a binary logistic regression model that any formula feedings during days 1 to 14 increased the risk of NEC 3.5 times. Thus, the early-life exposure to FM, in a critical window of transition from intrauterine to extrauterine nutrition, rather than a specific daily volume or percentage of MM within the total enteral feedings, increases the risk of NEC in VLBW infants [13,28]. This is in accordance with our results where no differences were found in the first day of enteral feeding and volume increases between control and NEC infants. In another study with a multivariable Cox analysis, infants who receive milk from their mothers during the first 5 days had lower risk of sepsis, NEC or mortality. This was also seen on day 6 to 10 if infants received more than 50% of their enteral nutrition as their mother’s milk [15]. These evidence are in accordance with our results, although we showed these protecting effects of MM in longitudinal and cross-sectional analysis.

There is insufficient evidence to assess human milk-derived fortifier versus cow’s milk-derived fortifier in preterm infants exclusively breastfed. The systematic Cochrane review by Premkumar and cols [9] reports that the evidence is low and indicates that in preterm infants fed exclusively on MM, fortifiers derived from MM, compared to those derived from cow’s milk, may not change the risk of NEC, mortality, food intolerance, and infection, nor improve growth. It concludes that well-designed randomized controlled trials are needed to assess short-term and long-term outcomes. However, in our study, FHM with cow’s milk significantly decreased the risk of NEC.

### Limitations and Strengths of the Study

As in many studies with such diverse feeding regimens, reverse causation can be a potential bias in this work. Particularly, in order to answer if prolonged fasting may be in the causal pathway to NEC development or if neonates were fasting due to the presence of NEC symptoms, we used the following distinct analysis: (1) We used panel data and we fit models for longitudinal analysis, such as generalized lineal models and mixed models, (2) We did a sensitivity analysis and stratified our analyses by fasting to evaluate the possibility of confounders, and (3) We performed cross-sectional analyses at different time-points (3, 7, and 14 days). Longitudinal and cross-sectional analysis showed the same results: fasting was associated with NEC and MM related to a decrease in the development of this pathology. Therefore, we believe that with these different approaches, we have decreased the possibility that the results presented may be highly related to reverse causation. Furthermore, fasting may not only be due to NEC but also related to vomit, PDA, shock, among others. Finally, we have to acknowledge the relatively small sample in this study.

## 5. Conclusions

The relevance of the study relies on the longitudinal and cross-sectional analysis showing that MM and FHM across time is related to decreased NEC together with antenal steroids administration. This further reinforces the notion that newborns receiving exclusive MM in the first two weeks after birth is key for reducing the odd for NEC development. This work includes recommendations regarding the use of MM in NICUs during the critical window of early feeding. Premature infant feeding practices should support the logistics for providing exclusive MM during this two-week crucial period. The results will warrant future studies to evaluate FHM in the protection of NEC development.

## Figures and Tables

**Table 1:** Clinical and demographic characteristics of the population.

Variable	Control (n = 92)	NEC (n = 100)
	Median, IQR (range)	Median, IQR (range)
Gestational age (weeks)	30, 28–32.5 (23.2–34.1)	30.3, 28.4–32.6 (25–34.4)
Day of NEC diagnosis		15, 5.2–25 (0–97)
Maternal age (years)	29, 22.2–34.7 (15–44)	28.5, 23–34 (14–46)
Birth weight (grams)	1270, 1005–1569 (580–2420)	1155, 901–1382 (560–2125)
	Control	NEC
	% (n)	% (n)
IUGR (yes)	22.8 (21)	35 (35)
Antenatal steroids (yes):	84.8 (78)	63.4 (64)
One dose	28.3 (26)	37.4 (37)
Two doses	47.7 (44)	22.2 (22)
More than two doses	8.8 (8)	4 (4)
Infant sex (Female)	51.1 (47)	49 (49)
Surfactant (yes)	35.9 (33)	31.3 (31)
PROM (yes)	30.4 (28)	32 (32)
Chorioamnionitis (yes)	7.6 (7)	14 (14)
Antenatal antibiotic exposure prior to birth(yes)	50 (46)	46 (46)
Maternal infection prior to birth (yes)	54.3 (50)	35 (35)
Early mechanical ventilation:		
CPAP (yes)	72.8 (67)	67 (67)
Endotracheal intubation (yes)	38 (35)	42 (42)

IUGR, Intrauterine Growth Restriction; PROM, Premature rupture of membranes; CPAP, Continuous Positive Airway Pressure.

**Table 2:** Birth weight in Control and necrotizing enterocolitis (NEC) infants.

Birth Weight Categories (g)	Controls % (n) (n = 92)	NEC % (n) (n = 100)
<1000	23 (21)	32 (32)
1001–1500	42 (39)	53 (53)
>1500	35 (32)	15 (15) *

* Chi-square *p* < 0.05, NEC vs. Control.

**Table 3:** Transversal feeding results for Control and NEC infants.

	Control % (*n*)	NEC % (*n*)	All % (*n*)	*p*-Value
Type of milk at birth				
Fasting	44.6 (41)	32 (32)	38 (73)	0.237
MM	27.2 (25)	30 (30)	28.6 (55)	
MM and DM	4.3 (4)	3 (3)	3.6 (7)	
MM and FM	5.4 (5)	2 (2)	3.6 (7)	
DM	3.3 (3)	5 (5)	4.2 (8)	
FM	5.4 (5)	12 (12)	8.9 (17)	
FHM	9.8 (9)	16 (16)	13 (25)	
Type of milk Day 3				
Fasting	16.7 (15)	16 (16)	16.3 (31)	0.806
MM	57.8 (52)	54 (54)	55.8 (106)	
MM and DM	11.1 (10)	10 (10)	10.5 (20)	
MM and FM	3.3 (3)	2 (2)	2.6 (5)	
DM	3.3 (3)	8 (8)	5.8 (11)	
FM	3.3 (3)	6 (6)	4.7 (9)	
FHM	4.4 (4)	4 (4)	4.2 (8)	
Type of milk Day 7				
Fasting	11.2 (10)	32.3 (32) *	22.3 (42)	0.013 *
MM	60.7 (54)	36.4 (36) *	47.9 (90)	
MM and DM	4.5 (4)	7.1 (7)	5.9 (11)	
MM and FM	11.2 (10)	9.1 (9)	10.1 (19)	
DM	3.4 (3)	5.1 (5)	4.3 (8)	
FM	3.4 (3)	4 (4)	3.7 (7)	
FHM	5.6 (5)	6.1 (6)	5.9 (11)	
Type of milk at Day 14				
Fasting	4.9 (4)	31.6 (31) *	19.6 (35)	<0.001 *
MM	53.1 (43)	37.8 (37) *	44.7 (80)	
MM and DM	6.2 (5)	6.1 (6)	6.1 (11)	
MM and FM	6.2 (5)	5.1 (5)	5.6 (10)	
DM	1.2 (1)	6.1 (6)	3.9 (7)	
FM	3.7 (3)	2 (2)	2.8 (5)	
FHM	24.7 (20)	11.2 (11) *	17.3 (31)	

* denotes a subset of feeding regimen categories whose column proportions between Control and NEC differ significantly from each other at the 0.05 level (z-test for independent proportions). MM: exclusive mother’s own milk, DM: exclusive donor milk, FM: exclusive preterm formula, MM and DM: combination of MM and DM; MM and FM: combination of MM and FM; and FHM: fortified milk.

**Table 4:** First day of enteral feeding and volume.

Variable	Control (*n* = 89)	NEC (*n* = 83)	*p*-Value
	Mean ± SD	Mean ± SD	
	(range)	(range)	
First day of enteral feeding (days)	1.4 ± 1.5	2.2 ± 3	0.052
	(0–10)	(0–19)	
Volume of first enteral feed (mL)	21.2 ± 18.7	15.5 ± 10.3	0.013 *
	(12.5–90)	(12.5–67)	
Increases (mL)	16.7 ± 12.7	28.9 ± 7.5	0.057
	(12.5–25)	(12.5–25)	
	NEC	SEC	
	(n = 62)	(n = 21)	
First day of enteral feeding (days)	1.9 ± 0.4	2.9 ± 3.2	0.035 *
	(0–19)	(0–12)	
Volume of first enteral feed (mL)	15.6 ± 10	14.9 ± 11	0.207
	(12.5–67)	(12.5–63)	
Increases (mL)	14.3 ± 4.4	13 ± 2.7	0.190
	(12.5–25)	(12.5–12.5)	

* *p* < 0.05, ECN vs. Control.

**Table 5:** Relationship between feeding strategies and NEC across three time points (3, 7, and 14 days): results from generalized linear model.

	Estimates (β)	95% CI	*p*-Value
Gestational age (weeks):			
25–28	Referral group	Referral group	Referral group
28–32	0.164	0.065–0.263	0.001
32–35	0.166	0.039–0.293	0.011
Birth weight (g):			
<1000	Referral group	Referral group	Referral group
1000–1500	−0.143	−0.042 to −0.338	0.005
>1500	−0.478	−0.618 to −0.338	<0.001
IUGR	−0.034	−0.123 to −0.054	0.447
Antenatal steroids:			
None	Referral group	Referral group	Referral group
1 dose	−0.188	−0.285 to −0.514	<0.001 *
2 doses	−0.414	−0.514 to −0.314	<0.001 *
Fasting	0.148	0.028–0.268	0.016 *
MM	−0.117	−0.227 to–0.007	0.038 *
DM	0.033	−0.069–0.134	0.527
FM	0.053	−0.67 to 0.173	0.386
FHM	−0.124	−0.243 to −0.006	0.039 *

Adjusted for gestational age at birth, birth weight, IUGR (Intrauterine Growth Restriction), and antenatal steroids. *p < 0.05.

**Table 6:** Relationship between feeding strategies and NEC adjusted for demographic variables: results from logistic regression model on days 3, 7, and 14 together.

	Adjusted Odds Ratio	95% CI	*p*-Value
Gestational age	1.729	1.247–2.398	0.001
Birth weight	0.995	0.992–0.997	<0.001
Antenatal steroids (2 doses)	0.157	0.052–0.474	0.001
Exclusive MM on day 7	0.148	0.044–0.501	0.002
Exclusive MM on day 14	0.048	0.010–231	<0.001
DM on day 14	0.043	0.02–0.491	0.013
FHM on day 14	0.027	0.004–0.179	<0.001
MM and DM on day 14	0.060	0.006–0.583	0.015

Ctrl versus NEC at day 3, day 7, and day 14, independently. Adjusted for gestational age, birth weight, IUGR, and antenatal steroids.

**Table 7:** Relationship between feeding strategies and NEC adjusted for demographic variables: results from logistic regression model on days 3, 7, and 14, independently.

	Adjusted Odds Ratio	95% CI	*p*-Value
MM on day 7	0.180	0.070–0.462	<0.001
MM and FM on day 7	0.220	0.057–0.847	0.028
MM on day 14	0.068	0.017–0.269	<0.001
FM on day 14	0.067	0.006–0.783	0.031
FHM on day 14	0.035	0.007–0.169	<0.001
MM and FM on day 14	0.101	0.014–0.705	0.021
MM and DM on day 14	0.072	0.011–0.461	0.005

Adjusted for gestational age, birth weight, infant sex, IUGR (Intrauterine Growth Restriction), and antenatal steroids.

**Table 8:** The onset of NEC at different time points in relation to fasting and type of milk.

	NEC Onset < 7 Days % (*n*)	NEC Onset > 7 Days % (*n*)	All % (*n*)	*p*-Value
Type of milk at birth				
Fasting	39.8 (49)	34.8 (24)	38 (73)	
MM	30.1 (37)	26.1 (18)	28.6 (55)	
MM and DM	3.3 (4)	4.3 (3)	3.6 (7)	
MM and FM	4.9 (6)	1.4 (1)	3.6 (7)	0.105
DM	5.7 (7)	1.4 (1)	4.2 (8)	
FM	8.1 (10)	10.1 (7)	8.9 (17)	
FHM	8.1 (10)	21.7 (15)*	13 (25)	
Type of milk Day 3				
Fasting	21. 5 (26)	7.2 (5)*	16.3 (31)	
MM	51.2 (62)	63.8 (44)	55.8 (106)	
MM and DM	12.4 (15)	7.2 (5)	10.5 (20)	0.093
MM and FM	3.3 (4)	1.4 (1)	2.6 (5)	
DM	5 (6)	7.2 (5)	5.8 (11)	
FM	3.3 (4)	7.2 (5)	4.7 (9)	
FHM	3.3 (4)	5.8 (4)	4.2 (8)	
Type of milk Day 7				
Fasting	70 (21)	15.9 (11) *	32.3 (32)	<0.001 *
MM	23.3 (7)	42 (29)	36.4 (36)	
MM and DM	0	10.1 (7)	7.1 (7)	
MM and FM	6.7 (2)	7.1 (7)	9.1 (9)	
DM	0	7.2 (5)	5.1 (5)	
FM	0	5.8 (4)	4 (4)	
FHM	0	8.7 (6)	6.1 (6)	
Type of milk Day 14				
Fasting	55.2 (16)	21.7 (15) *	31.6 (31)	0.015 *
MM	27.6 (8)	42 (29) *	37.8 (37)	
MM and DM	3.4 (1)	7.2 (5)	6.1 (6)	
MM and FM	3.4 (1)	5.8 (4)	5.1 (5)	
DM	10.3 (3)	4.3 (3)	6.1 (6)	
FM	0	2.9 (2)	2 (2)	
FHM	0	15.9 (11) *	11.2 (11)	

Each * denotes a subset of feeding regimen categories whose column proportions differ significantly from each other at the 0.05 level (z-test for independent proportions). MM: exclusive human milk, DM: donor milk, FM: preterm formula, FHM: fortified milk. One missing value.

## Data Availability

Upon request from the author.

## Appendix A

**Table A1:** Statistical test results for baseline clinical characteristics in Control and NEC groups.

Variable	*p*-Value
Gestational age (weeks)	0.511
Maternal age (years)	0.854
Birth weight (grams)	0.065
IUGR (yes)	0.080
Antenatal steroids (yes)	<0.001 *
Infant sex (Female)	0.885
Surfactant (yes)	0.542
PROM (yes)	0.877
Chorioamnionitis (yes)	0.173
Antenatal antibiotic (yes)	0.665
Maternal infection (yes)	0.009 *
Early mechanical ventilation:	
CPAP (yes)	0.433
Endotracheal intubation (yes)	0.659

* *p* < 0.05 between Control and NEC infants. IUGR, Intrauterine Growth Restriction; PROM, Premature rupture of membranes; CPAP, Continuous Positive Airway Pressure.

**Table A2:** Risk factors for NEC in Control and NEC infants.

Variable	Control (*n* = 92)	NEC (*n* = 100)	*p*-Value
	% (*n*)	% (*n*)	
Treatment (yes):			
Histamine H2 receptor block	10.9 (10)	9 (9)	0.810
Proton pump inhibitor	25 (23)	7 (7)	0.001 *
PDA (yes)	28.3 (26)	31 (31)	0.752
Sepsis (yes):			0.103
Early-onset	22 (20)	34 (34)	
Late-onset	4.4 (4)	7 (7)	
IRDS (yes)	57.6 (53)	38 (38)	0.009 *

PDA, Patent Ductus Arteriosus; IRDS, Infant Respiratory Distress Syndrome. ** p <0.05.*

**Table A3:** Relationship between feeding strategies and NEC across three time-points (3, 7 and 14 days): results from Mixed Model.

	Estimates (β)	95% CI	*p*-Value
Gestational age (weeks):			
25–28	−0.165	−0.295 to −0.036	0.012 *
28–32	−0.002	−0.104 to 0.101	0.973
32–35	Referral group	Referral group	Referral group
Birth weight (g):			
<1000	0.478	0.335–0.62	<0.001 *
1000–1500	0.334	0.23–0.438	<0.001 *
>1500	Referral group	Referral group	Referral group
IUGR (no)	0.034	−0.055–0.124	0.75
Antenatal steroids:			
None	Referral group	Referral group	Referral group
1 dose	−0.188	−0.287 to −0.0885	0.0002 *
2 doses	−0.414	−0.516 to −0.313	<0.0001 *
Fasting (no)	−0.148	−0.267 to −0.026	0.017 *
MM (no)	0.117	0.005–0.229	0.040 *
DM (no)	−0.032	−0.136–0.070	0.532
FM (no)	−0.053	−0.174 to 0.067	0.392
FHM (no)	0.124	−0.004–0.244	0.042 *

Adjusted for gestational age at birth, birth weight, IUGR (Intrauterine Growth Restriction), and antenatal steroids. ** p <* 0.05.

**Table A4:** Relationship between feeding strategies and NEC adjusted for demographic variables: results from Logistic regression model on days 3, 7, and 14 together.

	Adjusted Odds Ratio	95% CI	*p*-Value
Exclusive MM on day 3	2.255	0.609–8.353	0.224
DM on day 3	0.685	0.081–5.822	0.729
FM on day 3	1.982	0.124–31.731	0.629
HFM on day 3	0.436	0.017–11.320	0.617
MM and FM on day 3	0.037	0.001–2.667	0.131
MM and DM on day 3	1.477	0.247–8.826	0.669
DM on day 7	1.189	0.120–11.772	0.882
FM on day 17	0.252	0.013–4.920	0.364
FHM on day 7	0.801	0.044–14.447	0.881
MM and FM on day 7	0.395	0.070–2.249	0.295
MM and DM on day 7	0.141	0.014–1.455	0.100
DM on day 14	0.322	0.018–5.649	0.438
MM and FM on day 14	0.112	0.009–0.583	0.082

Ctrl versus NEC at day 3, day 7, and day 14, independently. Adjusted for gestational age, birth weight, IUGR, and antenatal steroids.

**Table A5:** Relationship between feeding strategies and NEC adjusted for demographic variables: results from Logistic regression model on days 3, 7, and 14, independently.

	Adjusted Odds Ratio	95% CI	*p*-Value
MM on day 3	0.850	0.347–2.080	0.721
DM on day 3	1.882	0.322–10.986	0.483
FM on day 3	1.153	0.0.198–6.702	0.874
FHM on day 3	0.568	0.100–3.229	0.524
MM and FM on day 3	0.394	0.042–3.676	0.413
MM and DM on day 3	0.696	0.201–2.419	0.569
DM on day 7	0.710	0.109–4.635	0.721
FM on day 7	0.291	0.046–1.842	0.190
FHM on day 7	0.270	0.051–1.440	0.125
MM and DM on day 7	0.299	0.065–1.374	0.121
DM on day 14	0.493	0.038–6.358	0.588

Adjusted for gestational age, birth weight, infant sex, IUGR (Intrauterine growth restriction), and antenatal steroids.
